# Comparative analysis of survival between elderly and non-elderly severe sepsis and septic shock resuscitated patients

**DOI:** 10.1590/S1679-45082015AO3313

**Published:** 2015

**Authors:** Henrique Palomba, Thiago Domingos Corrêa, Eliézer Silva, Andreia Pardini, Murillo Santucci Cesar de Assuncao

**Affiliations:** 1 Hospital Israelita Albert Einstein, São Paulo, SP, Brazil.

**Keywords:** Aged, Sepsis, Shock, septic, Shock, Resuscitation, Multiple organ failure, Fluid therapy, Vasoconstrictor agents

## Abstract

**Objective:**

To compare outcomes between elderly (≥65 years old) and non-elderly (<65 years old) resuscitated severe sepsis and septic shock patients and determine predictors of death among elderly patients.

**Methods:**

Retrospective cohort study including 848 severe sepsis and septic shock patients admitted to the intensive care unit between January 2006 and March 2012.

**Results:**

Elderly patients accounted for 62.6% (531/848) and non-elderly patients for 37.4% (317/848). Elderly patients had a higher APACHE II score [22 (18-28) *versus* 19 (15-24); p<0.001], compared to non-elderly patients, although the number of organ dysfunctions did not differ between the groups. No significant differences were found in 28-day and in-hospital mortality rates between elderly and non-elderly patients. The length of hospital stay was higher in elderly compared to non-elderly patients admitted with severe sepsis and septic shock [18 (10-41)* versus* 14 (8-29) days, respectively; p=0.0001]. Predictors of death among elderly patients included age, site of diagnosis, APACHE II score, need for mechanical ventilation and vasopressors.

**Conclusion:**

In this study population early resuscitation of elderly patients was not associated with increased in-hospital mortality. Prospective studies addressing the long-term impact on functional status and quality of life are necessary.

## INTRODUCTION

Severe sepsis and septic shock are major reasons for intensive care unit (ICU) admission worldwide and they are associated with high morbidity and mortality rates, despite intense efforts towards early diagnosis and treatment.^(^ ^1^ ^-^ ^3^ ^)^

Rivers et al.^(^ ^4^ ^)^ proposed the concept of early goal-directed therapy for the treatment of severe sepsis and septic shock patients in 2001. This principle has been incorporated in the Surviving Sepsis Campaign International Guidelines for Management of Severe Sepsis and Septic Shock.^(^ ^5^ ^) ^ Accordingly, early identification, broad spectrum antibiotic administration and hemodynamic stabilization have been the cornerstone of severe sepsis and septic shock management.^(^ ^6^ ^)^

The number of elderly patients (age ≥65 years old according to the World Health Organization) with severe sepsis and septic shock has been increasing steadily.^(^ ^7^ ^)^ This population of elderly patients is characterized by an increased prevalence of chronic illness, comorbidities, frailty and functional impairment.^(^ ^8^ ^,^ ^9^ ^)^ Nevertheless, while recent evidence has demonstrated that elderly patients submitted to complex therapeutic interventions during hospitalization showed benefits in long-term survival,^(^ ^10^ ^)^ observational studies have shown that increased age is an independent predictor of death among septic and non-septic patients.^(^ ^11^ ^-^ ^13^ ^)^

We postulated that elderly patients with severe sepsis or septic shock resuscitated following the Surviving Sepsis Campaign Guidelines have similar short-term mortality rates when compared to non-elderly patients with severe sepsis or septic shock.

## OBJECTIVE

To perform a retrospective, single-center cohort study to compare the outcomes between elderly (≥65 years) and non-elderly (<65 years) severe sepsis and septic shock resuscitated patients and to determine the main predictors of death among elderly patients.

## METHODS

This study was approved and the informed consent waived by the *Hospital Israelita Albert Einstein* Institutional Review Board (protocol 716,880 and CAAE: 32786114. 1.0000.0071). This study was conducted in a 41-bed medical-surgical ICU of a tertiary care at a private hospital in São Paulo, Brazil.

### Patients

According to the institutional protocol for severe sepsis and septic shock resuscitation, all patients admitted to the emergency department or those in hospital who had been seen by the rapid response team and fulfilled the criteria for severe sepsis and septic shock were admitted to the ICU. All adult patients with severe sepsis or septic shock admitted to the ICU between January 2006 and December 2012 were included in this study. A case manager followed these patients until hospital discharge and their data were recorded.

The criteria for admission to hospital floor, intermediary care and ICU for patients with no diagnosis of severe sepsis and septic shock was based on the clinical judgment of the attending physician. However, severe sepsis and septic shock patients coming from the emergency department, or those who had been screened by the rapid response team were necessarily admitted to the ICU.

### Definitions

The American College of Chest Physicians/Society of Critical Care Medicine definitions were used and sepsis was defined as infection plus two or more systemic inflammatory response syndrome (SIRS) criteria: temperature >38°C or <36°C, heart rate >90/minute, respiratory rate >20/minute or PaCO_2_ <32mmHg, white blood cell count >12,000cells/mL or <4,000cells/mL (or >10% band forms).^(^ ^14^ ^)^ Severe sepsis was defined as sepsis associated with organ dysfunction, including mental status changes, systolic blood pressure <90mmHg or mean arterial pressure (MAP) <65mmHg, serum creatinine >2.0mg/dL or diuresis <0.5mL/kg/h, total bilirubin >2.0mg/dL, platelet count <100,000 cells/mm^3^, arterial lactate >1.5 time the normal value, INR >1.5 or TTPa >60 seconds and relationship between arterial oxygen partial pressure and fraction of inspired oxygen (PaO_2_/FiO_2_) <300.

Septic shock was defined as sepsis-induced hypotension (systolic blood pressure <90mmHg or mean arterial blood pressure <65mmHg or a drop of >40mmHg in the absence of another cause of hypotension) despite adequate fluid resuscitation. Elderly patients were defined according to the World Health Organization as those aged ≥65 years.

### Early goal-directed therapy

All patients were resuscitated following the institutional protocol for severe sepsis and septic shock. The onset of treatment was defined as the time of severe sepsis and septic shock diagnosis. Once a patient was diagnosed with severe sepsis or septic shock, the 6-hour resuscitation bundle was initiated. This included blood sampling with measurement of arterial lactate level, collection of blood cultures before antibiotics administration, broad-spectrum antibiotics administration within 1 hour of the onset and a fluid load with crystalloids (20mL/kg) or equivalent doses of colloids.^(^ ^5^ ^)^

The early goal-directed therapy was applied to patients with severe sepsis associated with arterial lactate levels ≥4.0mmol/L or those who remained hypotensive (systolic blood pressure <90mmHg or MAP <65mmHg) despite fluid resuscitation with crystalloids (20mL/kg) or equivalent doses of colloids. After the diagnosis of severe sepsis or septic shock, the following therapeutic goals were targeted during the first 6-hours of resuscitation: central venous pressure between 8 and 12mmHg (12 to 15mmHg in mechanically ventilated patients), MAP ≥65mmHg, central venous oxygen saturation (SvcO_2_) or mixed venous (SvO_2_) ≥70% and 65%, and diuresis ≥0.5mL/kg/h.

### Variables collected

Demographic data, number of comorbidities, location before ICU admission, number of new organ dysfunctions at severe sepsis and septic shock diagnosis, source of infection, Acute Physiology and Chronic Health Evaluation II (APACHE) score,^(^ ^15^ ^) ^ need for vasopressors, invasive mechanical ventilation, amount of fluids administered, in-hospital and ICU length of stay, in-hospital and mortality at day 28 were collected.

### Statistical analysis

Categorical variables were presented as absolute and relative frequencies. Continuous variables were presented as mean and standard deviation (SD) when normally distributed and as median and interquartile range (IQR) when not normally distributed (tested by the Kolmogorov-Smirnov test).

Patients were divided into two groups according to the age: elderly patients (≥65 years) and non-elderly patients (<65 years). Categorical data were compared between elderly and non-elderly patients with the χ^2^ test or Fisher’s exact test when appropriate. Continuous data were compared with the independent *t* test when normally distributed and with the Mann-Whitney U test in the case of non-normal distribution.

A univariate logistic regression analysis was first performed to identify which factors or predictors were associated with in-hospital mortality in all study patients and then only among the elderly patients. Predictors that showed a p value ≤0.20 in the univariate analysis were entered into the multivariate analysis. A multivariate logistic regression analysis with a backward elimination procedure was undertaken to obtain an adjusted *odds ratio* (OR) along with 95% confidence interval (95%CI) and define which variables were independently associated with in-hospital mortality between all study patients and then only among the elderly patients. Statistical tests were 2-sided and p<0.05 was considered statistically significant. Statistical analyses were performed using IBM™ Statistical Package for the Social Science (SPSS™) version 20.0 for Windows.

## RESULTS

### Patients

This analysis included 848 patients admitted to the ICU with severe sepsis or septic shock. Elderly patients accounted for 62.6% (531/848) of patients and non-elderly patients for 37.4% (317/848) of patients (Table 1). The median (IQR) age was, respectively, for elderly and nonelderly patients, 80 years (73-86) and 51 years (40-59), with p<0.001.

**Table 1:** Baseline characteristics of study participants

Characteristic	<65 years 317 (37.4%) n (%)	≥65 years 531 (62.6%) n (%)	p value
Male sex	178 (56.2)	310 (58.4)	0.566
Underlying disease			
Systemic hypertension	89 (28.1)	280 (52.9)	<0.001
*Diabetes mellitus*	69 (21.8)	173 (32.7)	0.001
Neoplasms	73 (23.0)	146 (27.6)	0.142
Congestive heart failure	12 (3.8)	73 (13.8)	<0.001
Coronary insufficiency	18 (5.7)	72 (13.6)	<0.001
COPD	7 (2.2)	67 (12.7)	<0.001
Chronic renal failure	10 (3.2)	44 (8.3)	0.003
Chronic renal failure RRT	12 (3.8)	30 (5.7)	0.255
Liver cirrhosis	60 (18.9)	20 (3.8)	<0.001
Solid organ transplantation	66 (20.8)	14 (2.6)	<0.001
HIV	3 (0.9)	1 (0.2)	0.151
Number of comorbid conditions			<0.001
0	85 (26.8)	75 (14.1)	
1	109 (34.4)	161 (30.3)	
2	80 (25.2)	168 (31.7)	
≥3	43 (13.6)	127 (23.9)	
Source of infection			
Respiratory tract	144 (45.4)	307 (57.8)	<0.001
Urinary tract	51 (16.1)	85 (16.0)	1.000
Abdominal	84 (26.5)	77 (14.5)	<0.001
Skin and soft tissue	9 (2.8)	25 (4.7)	0.208
Others	12 (3.8)	20 (3.8)	1.000
Bloodstream	13 (4.1)	10 (1.9)	0.078
Unknown	4 (1.3)	7 (1.3)	1.000
Site of diagnosis, n (%)			
Emergency department	155 (48.9)	270 (50.8)	0.619
Hospital floor	107 (33.8)	104 (19.6)	<0.001
Intermediary care	18 (5.7)	93 (17.5)	<0.001
Intensive care unit	33 (10.4)	61 (11.5)	0.653
Other	4 (1.3)	3 (0.6)	0.434

p values are given by χ^2^ test or Fisher’s exact test for binary variables and Unpaired test for continuous variables. COPD: chronic obstructive pulmonary disease; HIV: human immunodeficiency virus; RRT: renal replacement therapy.

Elderly patients were more likely to have systemic hypertension (52.9% *versus* 28.1%; p<0.001), diabetes (32.7% *versus* 21.8%; p=0.001), ischemic heart disease (13.6% *versus* 5.7%; p<0.001), congestive heart failure (13.8% *versus *3.8%; p<0.001), chronic renal failure (8.3% *versus* 3.2%; p=0.003) and chronic obstructive pulmonary disease (12.7% *versus* 2.2%; p<0.001) when compared to non-elderly patients. Solid organ transplantation (20.8% *versus* 2.6%; p<0.001) and liver cirrhosis (18.9% *versus *3.8%; p<0.001) were more frequent in non-elderly patients compared to elderly patients (Table 1).

The main source of infection in elderly and non-elderly patients was the respiratory tract (57.8% *versus* 45.4%, for elderly and non-elderly patients; p<0.001) while intra-abdominal infections were more common in non-elderly patients.

### Site of diagnosis

The most common patient location at severe sepsis and septic shock diagnosis was the emergency department, with no difference between elderly and non-elderly patients (50.8% *versus* 48.9%; p=0.619) (Table 1).

A large proportion of non-elderly patients were diagnosed on the hospital floor (33.8% *versus* 19.6%, for non-elderly and elderly patients; p<0.001), while intermediary care was the most frequent site of diagnosis for elderly patients in comparison to non-elderly patients (17.5% *versus* 5.7%; p<0.001) (Table 1).

### Clinical presentation

The frequency of severe sepsis (43.3% *versus* 44.5%, for elderly and non-elderly patients; p=0.783) and septic shock (56.7% *versus* 55.5%, for elderly and non-elderly patients; p=0.783) did not differ between the groups (Table 2). Elderly patients had a higher median (IQR) APACHE II score [22 (18-28) *versus* 19 (15-24); p<0.001] compared to non-elderly patients, although the median number of new organ dysfunctions did not differ between the groups (p=0.829).

**Table 2:** Clinical presentation of study patients

Characteristic	<65 years 317 (37.4%)	≥65 years 531 (62.6%)	p value
APACHE II score, median [IQR]	19 [15-24]	22 [18-28]	<0.001
Arterial lactate (mmol/L), median [IQR]	2.4 [1.3-4.1]	2.2 [1.4-3.6]	0.285
Severe sepsis, n (%)	141 (44.5)	230 (43.3)	0.775
Septic shock, n (%)	176 (55.5)	301 (56.7)	
Clinical presentation, n (%)			
Hypotension	229 (72.2)	384 (72.3)	1.000
Lactate ≥4.0mmol/L	86 (27.7)	101 (19.7)	0.008
Number of organ dysfunctions, median [IQR]	2 [2-4]	3 [2-3]	0.829
Organ dysfunction, n (%)			
Circulatory	230 (72.6)	361 (68.0)	0.165
Respiratory	181 (57.1)	325 (61.2)	0.248
Renal	136 (42.9)	205 (38.6)	0.220
CNS	92 (29.0)	181 (34.1)	0.130
Hepatic	19 (6.0)	17 (3.2)	0.055
Metabolic	106 (33.4)	177 (33.3)	1.000
Hematologic	93 (29.3)	130 (24.5)	0.126

p values are given by χ^2^ test for binary variables and Mann-Whitney U test for continuous variables; APACHE II: Acute Physiology and Chronic Health Evaluation II (the score can range from zero to 71, with higher scores indicating more severe illness); IQR: interquartile range; CNS: central nervous system.

### Administered treatments

Compliance with the institutional protocol for severe sepsis and septic shock resuscitation has been published elsewhere.^(^ ^3^ ^)^ The elderly patients received less fluid [median (IQR)] during the initial 6-hours of resuscitation than the non-elderly patients [1.8 (1.0 to 2.5)* versus* 2.0 (1.4 to 3.0) liters, for elderly and non-elderly patients; p=0.001]. The need for vasopressors (58.8% *versus* 58.7, for elderly and non-elderly patients; p=0.981) and mechanical ventilation (38.4% *versus* 38.2%, for elderly and non-elderly patients; p=0.943) did not differ between the groups.

### Outcomes

In-hospital mortality and mortality at day 28 did not differ between elderly and non-elderly severe sepsis and septic shock patients (Table 3). The median (IQR) length of hospital stay was higher in elderly compared to non-elderly patients admitted with severe sepsis [15 (8-34) *versus* 12 (6-24) days; p=0.027] or septic shock [21 (11-47) *versus* 18 (9-36) days; p=0.016]. The median length of ICU stay did not differ between elderly and non-elderly severe sepsis and septic shock patients.

**Table 3:** Mortality rates and length of intensive care unit and hospital stay

Outcomes	<65 years 317 (37.4%)	≥65 years 531 (62.6%)	p value
Severe sepsis			
Mortality day 28, n (%)	13/135 (9.6)	33/217 (15.2)	0.146
In-hospital mortality, n (%)	20/141 (14.2)	47/230 (20.4)	0.164
Length of ICU stay (days), median [IQR]	3 [2-7]	3 [1-9]	0.583
Length of hospital stay (days), median [IQR]	12 [6-24]	15 [8-34]	0.027
Septic shock			
Mortality day 28, nº (%)	63/166 (38.0)	101/290 (34.8)	0.543
In-hospital mortality, nº (%)	70/176 (39.8)	134/301(44.5)	0.338
Length of ICU stay (days), median [IQR]	5 [2-12]	6 [3-13]	0.146
Length of hospital stay (days), median [IQR]	18 [9-36]	21 [11-47]	0.016
Severe sepsis and septic shock			
Mortality day 28, n (%)	76/301 (25.2)	134/507 (26.4)	0.740
In-hospital mortality, n (%)	90/317 (28.4)	181/531 (34.1)	0.094
Length of ICU stay (days), median [IQR]	4 [2-10]	5 [2-11]	0.141
Length of hospital stay (days), median [IQR]	14 [8-29]	18 [10-41]	0.001

p values are given by χ^2^ test for binary variables and Mann-Whitney U test for continuous variables. ICU: intensive care unit; IQR: interquartile range.

### Predictors of death

The univariate and multivariate logistic regression analysis addressing the predictors of death in all septic patients and only among the elderly patients are presented in tables 4 and 5, respectively.

The site of diagnosis, the presence of liver cirrhosis, APACHE II score, the arterial blood lactate level, the number of organ dysfunction and the need for mechanical ventilation were independently associated with increased risk of in-hospital death among severe sepsis and septic shock patients. Increased age, the site of diagnosis, APACHE II score, the need for mechanical ventilation and vasopressor administration were independently associated with increased risk of in-hospital death among elderly patients.

## DISCUSSION

This study demonstrated that in-hospital and 28-day mortality rates were not different between elderly and non-elderly patients submitted to early goal-directed therapy for the treatment of severe sepsis and septic shock. However, increased age was an independent predictor of in-hospital death among elderly patients. Therapeutic goals included in the first 6 hours of resuscitation from the initial diagnosis of severe sepsis and septic shock were achieved similarly in both groups. Nevertheless, less fluid for hemodynamic stabilization was administered to the elderly patients.

Traditionally, elderly patients receive less intensive treatment compared to non-elderly patients, probably due to the possibility of deleterious effects of an aggressive therapy and fear of fluid overload.^(^ ^16^ ^)^ Recently, increased acceptance of complex ICU interventions in older patients was associated with greater intensity of treatment and improved survival.^(^ ^10^ ^)^ This is perhaps the result of increased experience with the care of elderly patients over the years and technical improvements such as protocols associated with hemodynamic monitoring tools and continuous renal replacement therapy, representing a true evolution in practice throughout the times. Our results confirm these findings, as the proportion of elderly patients receiving mechanical ventilation and vasopressors was not different from that in younger patients.

Our findings confirm the tolerance of elderly patients to an early goal-directed therapy algorithm for severe sepsis and septic shock resuscitation, with no differences in mortality compared to non-elderly patients. These results have important clinical implications, since there is an increasing demand for ICU admission of elderly patients, which is often associated with high costs and limited availability of ICU beds worldwide.^(^ ^7^ ^,^ ^17^ ^,^ ^18^ ^)^ Our results support the concept that ICU admission and early goal-directed therapy implementation should not be denied to elderly patients showing severe sepsis and septic shock.

The impact of age itself on higher mortality rates due to sepsis is not uniformly observed in epidemiological investigations.^(^ ^11^ ^,^ ^19^ ^,^ ^20^ ^)^ Other retrospective analysis demonstrated that age was associated with a significantly increased risk of death in elderly patients with severe sepsis or septic shock.^(^ ^11^ ^,^ ^18^ ^)^ Nonetheless, these studies rely on administrative databases for sepsis diagnosis, which may be inaccurate and lacking important aspects such as adherence to the proposed treatment. Similarly, in our study, after adjustments for baseline patient’s characteristics in a multivariable logistic regression model, age was independently associated with increased risk of in-hospital mortality in elderly patients with severe sepsis or septic shock.

Most studies addressing mortality in septic patients have focused on short-term endpoints.^(^ ^4^ ^,^ ^12^ ^)^ Few observational studies have addressed the long-term prognosis of elderly severe sepsis and septic shock patients submitted to early goal-directed therapy. Lemay et al. reported a 1-year mortality rate of 31% in elderly patients with severe sepsis.^(^ ^20^ ^) ^Nevertheless, adherence to specific therapeutic goals besides antibiotics administration was not reported and the study relied on administrative database for sepsis diagnosis. Similarly, Wang et al. described a 1-year mortality rate of 23% in a population of adults aged 45 or older, with sepsis defined as hospitalization or treatment in the emergency department for a serious infection with the presence of two or more systemic inflammatory response criteria, with no mention of ICU patients with severe sepsis or septic shock.^(^ ^21^ ^)^ Recently, it was shown that long-term survival in elderly patients with circulatory failure (including sepsis) is poor, with mortality rates of 92 and 97% after 6 and 12 months, respectively.^(^ ^13^ ^)^ These findings support the hypothesis that excess long-term mortality persists among those suffering from sepsis, probably because sepsis triggers an independent pathophysiological process leading to early death.

Respiratory infections accounted for the most sepsis cases in elderly patients, whereas abdominal infections were the most common cause in younger patients, a finding which has not been confirmed by other authors, where respiratory infection was the major source of infection in both elderly and non-elderly patients.^(^ ^11^ ^)^ A possible explanation for this interesting finding is the greater incidence of liver cirrhosis and solid organ transplantation in younger patients, probably reflecting a high proportion of patients with spontaneous bacterial peritonitis.

Our study has limitations. First, we were unable to evaluate the functional status before and after ICU discharge. Functional status has been related to pre-existing underlying factors, and it plays a greater role than chronological age in the outcome of elderly patients with severe sepsis and septic shock.^(^ ^22^ ^)^ Second, this was a single center and retrospective study, which potentially limits the generalizability of our findings. Lastly, we used 65 as the cut-off age following the definition of elderly by World Health Organization. However, as pointed out earlier, the chronological age is not always representative of the functional condition of the patients.

## CONCLUSION

In this study population of severe sepsis and septic shock patients, early resuscitation of elderly patients was not associated with increase in mortality. Elderly patients with severe sepsis or septic shock may benefit from aggressive resuscitation and advanced treatment modalities. However, prospective studies are warranted to address long-term impact of resuscitation maneuvers on functional status and quality of life.

**Table 4:** Univariate and multivariate logistic regression analysis addressing the main risk factors for in-hospital mortality including 848 severe sepsis and septic shock patients

Characteristics	Univariate analysis			Multivariate analysis		
	OR	95%CI	p value	OR	95%CI	p value
Male sex	0.82	0.61-1.10	0.182	0.68	0.48-0.97	0.032
Site of diagnosis						
Emergency department	1.00					
Hospital floor	3.10	2.14-4.48	<0.001	2.74	1.78-4.22	<0.001
Intermediary care	4.93	3.15-7.71	<0.001	4.39	2.63-7.35	<0.001
Intensive care unit	5.78	3.59-9.31	<0.001	3.64	2.04-6.50	<0.001
Liver cirrhosis	2.62	1.65-4.18	<0.001	2.13	1.22-3.72	0.008
Urinary tract infection	0.30	0.18-0.49	<0.001	0.40	0.21-0.75	0.040
APACHE II score	1.11	1.08-1.13	<0.001	1.08	1.05-1.11	<0.001
Arterial lactate	1.18	1.12-1.25	<0.001	1.14	1.06-1.22	<0.001
Number of organ dysfunction	1.56	1.38-1.76	<0.001	1.21	1.04-1.40	0.012
Mechanical ventilation	4.94	3.62-6.73	<0.001	1.89	1.29-2.78	0.001

APACHE II: Acute Physiology and Chronic Health Evaluation II (the score can range from zero to 71, with higher scores indicating more severe illness). OR: *odds ratio;* 95%CI: 95% confidence interval.

**Table 5:** Univariate and multivariate logistic regression analysis addressing the main risk factors for in-hospital mortality among the 531 elderly (≥65 years) patients

Characteristics	Univariate analysis			Multivariate analysis		
	OR	95%CI	p value	OR	95%CI	p value
Age, years	1.04	1.02-1.07	<0.001	1.04	1.01-1.07	0.003
Male sex	0.69	0.48-1.00	0.048	0.61	0.40-0.94	0.023
Site of diagnosis						
Emergency department	1.00					
Hospital floor	2.71	1.67-4.39	<0.001	2.40	1.37-4.20	0.002
Intermediary care	3.82	2.31-6.30	<0.001	3.71	2.07-6.65	<0.001
Intensive care unit	4.12	2.31-7.37	<0.001	3.07	1.52-6.20	0.002
Systemic hypertension	0.63	0.44-0.90	0.012	0.56	0.36-0.86	0.009
*Diabetes mellitus *	0.58	0.39-0.87	0.008	0.58	0.36-0.94	0.025
Abdominal infection	0.69	0.40-1.18	0.174	0.50	0.27-0.95	0.034
APACHE II score	1.09	1.06-1.12	<0.001	1.06	1.03-1.10	<0.001
Mechanical ventilation	5.11	3.47-7.52	<0.001	2.18	1.36-3.52	0.001
Vasopressor administration	3.25	2.17-4.85	<0.001	1.98	1.24-3.18	0.005

APACHE II: Acute Physiology and Chronic Health Evaluation II (the score can range from zero to 71, with higher scores indicating more severe illness). OR: *odds ratio;* 95%CI: 95% confidence interval.
