# Supplementary material for: Comparative analysis of survival between elderly and non-elderly severe sepsis and septic shock resuscitated patients
Source: Einstein (Sao Paulo). 2015 Jul-Sep;13(3):357–63. doi: 10.1590/S1679-45082015AO3313 (PMC4943779; doi:10.1590/S1679-45082015AO3313)
Supplement: Translation

# Translation: Análise comparativa da sobrevida de idosos e não idosos com sepse grave ou choque séptico ressuscitados

**Authors:** Henrique Palomba, Thiago Domingos Corrêa, Eliézer Silva, Andreia Pardini, Murillo Santucci Cesar de Assuncao

**Affiliations:** 1 Hospital Israelita Albert Einstein, São Paulo, SP, Brasil.

**Keywords:** Idoso, Sepse, Choque séptico, Choque, Ressuscitação, Insuficiência de múltiplos órgãos, Hidratação, Vasoconstritores

## Abstract

**Objetivo:**

Comparar os resultados obtidos com a ressuscitação de idosos (≥65 anos) e não idosos (<65 anos) com sepse grave ou choque séptico e determinar os preditores de óbito em pacientes idosos.

**Métodos:**

Estudo de coorte retrospectivo com 848 pacientes com sepse grave ou choque séptico admitidos na unidade de terapia intensiva entre janeiro de 2006 e março de 2012.

**Resultados:**

Pacientes idosos representaram 62,6% (531/848) e não idosos 37,4% (317/848) dos pacientes. Pacientes idosos apresentaram maior escore APACHE II [22 (18-28)* versus* 19 (15-24); p<0,001] em comparação com pacientes não idosos, embora o número de disfunções orgânicas não tenha sido diferente entre os grupos. Não se observaram diferenças significativas na mortalidade hospitalar e em 28 dias entre pacientes idosos e não idosos, embora o tempo de internação hospitalar tenha sido superior nos pacientes idosos, em comparação com não idosos [18 (10-41) *versus* 14 (8-29) dias, respectivamente; p=0,0001]. Foram preditores de óbito entre pacientes idosos a idade, o local do diagnóstico, o escore APACHE II e a necessidade de ventilação mecânica e vasopressores.

**Conclusão:**

A ressuscitação de pacientes idosos com sepse grave ou choque séptico não associou-se ao aumento de mortalidade hospitalar. Estudos prospectivos são necessários para avaliação do impacto a longo prazo no estado funcional e qualidade de vida dos pacientes idosos ressuscitados.

## INTRODUÇÃO

Sepse grave e choque séptico são os principais motivos para internação em unidades de terapia intensiva (UTI) em todo mundo e também estão associadas a altas taxas de mortalidade e morbidade, apesar dos intensos esforços para diagnóstico e tratamento precoces.^(^ ^1^ ^-^ ^3^ ^)^

Em 2001, o conceito de terapia precoce guiada por metas para tratamento de sepse grave e choque foi proposto por Rivers et al.^(^ ^4^ ^) ^Esse conceito foi incorporado na *Surviving Sepsis Campaign International Guidelines for Management of Severe Sepsis and Septic Shock*.^(^ ^5^ ^) ^Os pilares do tratamento de sepse grave e choque séptico são identificação adequada e precoce, administração de amplo espectro de antibióticos e estabilização hemodinâmica.^(^ ^6^ ^)^

O número de pacientes idosos (≥65 anos, de acordo com a Organização Mundial da Saúde) com sepse grave e choque séptico tem crescido exponencialmente.^(^ ^7^ ^)^ Essa população de idosos é caracterizada por aumento da prevalência de doenças crônicas, comorbidades, debilidade e comprometimentos funcionais.^(^ ^8^ ^,^ ^9^ ^)^ Todavia, enquanto evidências recentes demonstram que pacientes idosos submetidos a intervenções terapêuticas complexas durante a hospitalização mostram benefícios na sobrevida de longo prazo,^(^ ^10^ ^)^ estudos observacionais mostram que o envelhecimento é um fator preditivo independente da mortalidade entre pacientes acometidos por sepse ou não.^(^ ^11^ ^-^ ^13^ ^)^

Acredita-se que a ressuscitação de pacientes idosos com sepse grave ou choque séptico, de acordo com a *Surviving Sepsis Campaign Guidelines*, apresenta taxas de mortalidade de curto prazo em comparação com paciente não idosos acometidos com sepse grave e choque séptico.

## OBJETIVO

Realizar estudo retrospectivo, coorte em centro único para comparar resultados de ressuscitação de pacientes idosos (≥65 anos) e não idosos (<65 anos) com sepse grave e choque séptico, e determinar os principais preditores de óbito entre pacientes idosos.

## MÉTODOS

Estudo aprovado pelo comitê de ética do Hospital Israelita Albert Einstein (protocolo 716.880 e o CAAE: 32786114.1.0000.0071), sendo dispensado o uso de Termo de Consentimento. O estudo foi conduzido em UTI de assistência terciária contendo 41 leitos de um hospital privado em São Paulo.

### Pacientes

Com base no protocolo institucional de ressuscitação por sepse grave e choque séptico, todos os pacientes internados na unidade de emergência ou hospitalizados que foram avaliados pela equipe emergencial e preenchiam os critérios para sepse grave ou choque séptico foram internados na UTI. Foram incluídos neste estudo todos os pacientes com sepse grave ou choque séptico admitidos na UTI entre janeiro de 2006 e dezembro de 2012. Os pacientes foram acompanhados por um pesquisador deste estudo até alta hospital e arquivamento de seus prontuários.

O critério para internação e encaminhamento (assistência ambulatorial, assistência intermediária e UTI) dos pacientes não diagnosticados com sepse grave ou choque séptico baseou-se no julgamento do médico responsável pelo atendimento. No entanto, pacientes com sepse grave e choque séptico originaram-se da unidade de emergência ou aqueles que, avaliados pela equipe emergencial, foram necessariamente internados na UTI.

### Definições

As definições do *American College of Chest Physicians*/*Society of Critical Care Medicine* foram utilizadas, e sepse foi definida como infecção contendo dois ou mais critérios da síndrome da resposta inflamatória sistêmica (SIRS): temperatura >38°C ou <36°C, frequência cardíaca >90/minuto, frequência respiratória >20/minuto ou PaCO_2_ <32mmHg, contagem de leucócitos >12.000cells/mL ou <4.000cells/mL (ou bastonetes >10%).^(^ ^14^ ^)^ A sepse grave foi definida como sepse associada a disfunção de órgão, incluindo mudanças no estado mental, pressão arterial sistólica <90mmHg ou pressão arterial média (PAM) <65mmHg, creatinina sérica >2,0mg/dL ou diureses <0,5mL/kg/h, bilirrubina total >2,0mg/dL, contagem de plaquetas <100.000 cells/mm^3^, lactado arterial >1,5 vez o valor normal, INR >1,5 ou TTPa >60 segundos e relação entre a pressão arterial de oxigênio e sua fração inspirada (PaO_2_/FiO_2_) <300.

O choque séptico foi definido como hipotensão induzida por sepse (pressão sanguínea sistólica <90mmHg ou PAM<65mmHg ou queda de >40mmHg na ausência de outra causa hipotensiva) apesar de ressuscitação com fluído adequada. Os pacientes idosos foram definidos de acordo com parâmetros da Organização Mundial da Saúde, ou seja, idade ≥65 anos.

### Terapia precoce guiada por metas

Todos os pacientes foram ressuscitados seguindo os protocolos institucionais para sepse grave e choque séptico. Uma vez diagnosticado com sepse grave ou choque séptico, foi iniciado pacote de 6 horas de ressuscitação. As ações incluíram amostragem sanguínea com medida do nível de lactato arterial, coleta de culturas sanguíneas anterior à administração de antibióticos, amplo espectro de administração de antibióticos até hora do início de carga de fluido com cristaloides (20mL/kg) ou doses equivalentes de coloides.^(^ ^5^ ^)^

A terapia precoce guiada por metas foi aplicada a pacientes com sepse grave associada com níveis de lactato arterial ≥4,0mmol/L ou aqueles que permaneceram hipotensivos (pressão sanguínea sistólica <90mmHg ou PAM<65mmHg), apesar da administração de fluidos com cristaloides (20mL/kg) ou doses equivalentes de coloides. Após o diagnóstico de sepse grave e choque séptico, as seguintes metas terapêuticas foram definidas durante a ressuscitação de 6 horas: pressão venosa central entre 8 e 12mmHg (12 a 15mmHg em pacientes mecanicamente ventilados), PAM ≥65mmHg, saturação venosa central de oxigênio (SvcO_2_) ou saturação venosa mista (SvO_2_) ≥70% e 65%, respectivamente e diurese ≥0,5mL/kg/h.

### Coleta de variáveis

No 28º dia, coletaram-se dados demográficos, número de comorbidades, localização antes da internação na UTI, número de novas disfunções em órgãos no diagnóstico de sepse grave e choque séptico, fonte da infecção, APACHE (*Acute Physiology and Chronic Health Evaluation II*),^(^ ^15^ ^)^ necessidade de vasopressores, ventilação mecânica invasiva, quantidade de fluido administrado, tempo de hospitalização e internação em UTI, internação e mortalidade.

### Análise estatística

As variáveis categóricas foram apresentadas por frequência absoluta e relativas. As variáveis contínuas foram apresentadas com medias e desvios padrões (DP), quando normalmente distribuídas, e como medianas e intervalo interquartil (IQR), quando não distribuídas normalmente (testado por meio do teste Kolmogorov-Smirnov).

Os pacientes foram separados em dois grupos de acordo com a idade: pacientes idosos (≥65 anos) e não idosos (<65 anos). Os dados categóricos foram comparados entre pacientes idosos e não idosos com teste χ^2^ ou teste exato de Fisher quando apropriado. Os dados contínuos foram comparados com teste *t* independente, quando distribuídos normalmente, e com teste U de Mann-Whitney, no caso de não normalidade na distribuição.

Primeiramente, realizou-se regressão logística univariada para identificar quais fatores ou preditores estavam associados com mortalidade na hospitalização em todos os pacientes estudados e, após, somente nos paciente idosos. Os preditores que mostraram valor de p ≤0,20 na análise univariada foram incluídos em análise multivariada. A regressão multivariada logística com procedimento *backward elimination* foi conduzida para obter *odds ratio* (OR) ajustado, juntamente do intervalo de confiança de 95% (IC95%), e definir quais variáveis são associadas independentemente com a mortalidade na hospitalização entre todos os pacientes do estudo e, após, somente entre os pacientes idosos. Os testes estatísticos foram teste bilateral e p<0,05 foi considerado estatisticamente significante. As análises estatísticas foram realizadas por meio do IBM^®^ *Statistical Package for the Social Science* (SPSS^®^), versão 20.0 para *Windows*.

## RESULTADOS

### Pacientes

Foram incluídos nesta análise 848 pacientes admitidos na UTI com sepse grave e choque séptico. Os pacientes idosos representaram 62,6% (531/848) e pacientes não idosos 37,4% (317/848) (Tabela 1). A média (IQR) de idade dos idosos e não idosos foi de 80 anos (73 a 86) e 51 (40 a 59), respectivamente, com p<0,001.

**Tabela 1:** Características de linha de base dos participantes do estudo

Características	<65 anos 317 (37,4%) n (%)	≥65 anos 531 (62,6%) n (%)	Valor de p
Sexo masculino	178 (56,2)	310 (58,4)	0,566
Doença de base			
Hipertensão sistêmica	89 (28,1)	280 (52,9)	<0,001
*Diabetes mellitus*	69 (21,8)	173 (32,7)	0,001
Neoplasias	73 (23,0)	146 (27,6)	0,142
Insuficiência cardíaca congestiva	12 (3,8)	73 (13,8)	<0,001
Insuficiência coronária	18 (5,7)	72 (13,6)	<0,001
DPOC	7 (2,2)	67 (12,7)	<0,001
Insuficiência renal crônica	10 (3,2)	44 (8,3)	0,003
Insuficiência renal crônica TSR	12 (3,8)	30 (5,7)	0,255
Cirrose hepática	60 (18,9)	20 (3,8)	<0,001
Transplante de órgão sólido	66 (20,8)	14 (2,6)	<0,001
HIV	3 (0,9)	1 (0,2)	0,151
Número de comorbidades			<0,001
0	85 (26,8)	75 (14,1)	
1	109 (34,4)	161 (30,3)	
2	80 (25,2)	168 (31,7)	
≥3	43 (13,6)	127 (23,9)	
Fonte de infecção			
Trato respiratório	144 (45,4)	307 (57,8)	<0,001
Trato urinário	51 (16,1)	85 (16,0)	1,000
Abdome	84 (26,5)	77 (14,5)	<0,001
Pele e tecido mole	9 (2,8)	25 (4,7)	0,208
Outros	12 (3,8)	20 (3,8)	1,000
Corrente sanguínea	13 (4,1)	10 (1,9)	0,078
Desconhecido	4 (1,3)	7 (1,3)	1,000
Local do diagnóstico, n (%)			
Unidade de emergência	155 (48,9)	270 (50,8)	0,619
Ambulatório	107 (33,8)	104 (19,6)	<0,001
Assistência intermediária	18 (5,7)	93 (17,5)	<0,001
Unidade de terapia intensiva	33 (10,4)	61 (11,5)	0,653
Outros	4 (1,3)	3 (0,6)	0,434

Valores de p são dados pelo teste χ^2^ ou teste exato de Fisher, para variáveis binárias, e teste não pareado, para variáveis contínuas. DPOC: doença pulmonar obstrutiva crônica; TSR: terapia de substituição renal; HIV: síndrome da imunodeficiência adquirida.

Pacientes idosos apresentaram maior probabilidade de apresentar hipertensão sistêmica (52,9% *versus* 28,1%; p<0,001), diabetes (32,7% *versus* 2,8%; p=0,001), doença cardíaca isquêmica (13,6% *versus* 5,7%; p<0,001), insuficiência cardíaca congestiva (13,8% *versus* 3,8%; p<0,001), insuficiência renal crônica (8,3% *versus* 3,2%; p=0,003) e doença pulmonar obstrutiva crônica (12,7% *versus* 2,2%; p<0,001) quando comparados a pacientes não idosos. O transplante de órgão sólido (20,8% *versus* 2,6%; p<0,001) e a cirrose hepática (18,9% *versus* 3,8%; p<0,001) foram mais frequentes em pacientes não idosos em comparação com pacientes idosos (Tabela 1).

A principal fonte de infecção em pacientes idosos e não idosos foi o trato respiratório (57,8% *versus* 45,4%, para idosos e não idosos; p<0,001), enquanto infecções intra-abdominal foram mais comuns em pacientes não idosos.

### Local do diagnóstico

O local mais comum do diagnóstico da sepse grave e do choque séptico foi a unidade de emergência, com diferença entre pacientes idosos e não idosos (50,8% *versus* 48,9%; p=0,619) (Tabela 1).

Uma grande proporção de pacientes não idosos foi diagnosticada na enfermaria (33,8% *versus* 19,6%, para paciente não idosos e idosos; p<0,001), porém o diagnóstico na assistência intermediária foi o local mais frequente para pacientes idosos comparados com pacientes não idosos (17,5% *versus* 5,7%; p<0,001) (Tabela 1).

### Apresentação clínica

A frequência da sepse grave (43,3% *versus* 44,5%, para idosos e não idosos; p=0,783) e choque séptico (56,7% *versus* 55,5%, para idosos e não idosos; p=0,783) não diferiu entre os grupos (Tabela 2). Os pacientes idosos tiveram uma alta média (IQR) no escore APACHE II [22 (18-28) *versus* 19 (15-24); p<0,001] comparado a pacientes não idosos, apesar de a média de novas disfunções de órgãos não diferir entre os grupos (p=0,829).

**Tabela 2:** Apresentação clínica dos participantes

Características	<65 anos 317 (37,4%)	≥65 anos 531 (62,6%)	Valor de p
Escore APACHE II, mediana [IQR]	19 [15-24]	22 [18-28]	<0,001
Lactato arterial (mmol/L), mediana [IQR]	2,4 [1,3-4,1]	2,2 [1,4-3,6]	0,285
Sepse aguda, n (%)	141 (44,5)	230 (43,3)	0,775
Choque séptico, n (%)	176 (55,5)	301 (56,7)	
Apresentação clínica, n (%)			
Hipotensão	229 (72,2)	384 (72,3)	1,000
Lactato ≥4,0mmol/L	86 (27,7)	101 (19,7)	0,008
Número de disfunções, mediana [IQR]	2 [2-4]	3 [2-3]	0,829
Disfunção de órgãos, n (%)			
Circulação	230 (72,6)	361 (68,0)	0,165
Respiratório	181 (57,1)	325 (61,2)	0,248
Renal	136 (42,9)	205 (38,6)	0,220
SNC	92 (29,0)	181 (34,1)	0,130
Hepático	19 (6,0)	17 (3,2)	0,055
Metabólico	106 (33,4)	177 (33,3)	1,000
Hematológica	93 (29,3)	130 (24,5)	0,126

Valores de p são dados por χ^2^, para variáveis binárias, e em teste U de Mann-Whitney, para variáveis contínuas. APACHE II: *Acute Physiology and Chronic Health Evaluation II *(escore pode variar de zero a 71, com alto escore indicando doença mais aguda); IQR: intervalo interquartil; SNC: sistema nervoso central.

### Administração de tratamentos

A aderência aos protocolos institucionais para ressuscitação de sepse grave e choque séptico tem sido largamente discutidas na literatura.^(^ ^3^ ^)^ Pacientes idosos receberam menos fluído [média (IQR)] durante as primeiras 6 horas de ressuscitação do que os pacientes não idosos [1,8 (1,0 a 2,5) *versus* 2,0 (1,4 a 3,0) L, para idosos e não idosos; p=0,001]. A necessidade de vasopressores (58,8% *versus* 58,7%, para idosos e não idosos; p=0,943) e ventilação mecânica (38,4% *versus* 38,2%, para idosos e não idosos; p=0,943) não apresentou diferença entre os grupos.

### Desfechos

A mortalidade hospitalar e mortalidade no 28º dia não se diferenciou entre idosos e não idosos com sepse grave e choque séptico (Tabela 3). A média (IQR) de permanência no hospital foi maior em idosos comparado com não idosos internados com sepse grave [15 (8-34) *versus* 12 (6-24) dias; p=0,027] ou choque séptico [21 (11-47) *versus* 18 (9-36) dias; p=0,016]. A média de permanência na UTI não diferiu entre idosos e não idosos com sepse aguda e choque séptico.

**Tabela 3:** Taxa de mortalidade e de permanência na unidade de terapia intensiva e hospitalar

Resultados	<65 anos 317 (37,4%)	≥65 anos 531 (62,6%)	Valor de p
Sepse aguda			
Mortalidade no 28^o^ dia, n (%)	13/135 (9,6)	33/217 (15,2)	0,146
Mortalidade hospitalar, n (%)	20/141 (14,2)	47/230 (20,4)	0,164
Permanência na UTI (dias), mediana [IQR]	3 [2-7]	3 [1-9]	0,583
Permanência no hospital (dias), mediana [IQR]	12 [6-24]	15 [8-34]	0,027
Choque séptico			
Mortalidade no 28^o^ dia, n (%)	63/166 (38,0)	101/290 (34,8)	0,543
Mortalidade hospitalar, n (%)	70/176 (39,8)	134/301(44,5)	0,338
Permanência na UTI (dias), mediana [IQR]	5 [2-12]	6 [3-13]	0,146
Permanência hospitalar (dias), mediana [IQR]	18 [9-36]	21 [11-47]	0,016
Sepse aguda e choque séptico			
Mortalidade no 28^o^ dia, n (%)	76/301 (25,2)	134/507 (26,4)	0,740
Mortalidade hospitalar, n (%)	90/317 (28,4)	181/531 (34,1)	0,094
Permanência na UTI (dias), mediana [IQR]	4 [2-10]	5 [2-11]	0,141
Permanência hospitalar (dias), mediana [IQR]	14 [8-29]	18 [10-41]	0,001

Valores de p são demonstrado por teste χ^2^, para variáveis binárias, e em teste U de Mann-Whitney, para variáveis continuadas. UTI: unidade de terapia intensiva; IQR: intervalo interquartil.

### Preditores de mortalidade

As análises de regressão logística multivariada e univariada abordando os preditores de mortalidade em todos pacientes com sepse e somente nos pacientes idosos estão apresentas nas tabelas 4 e 5, respectivamente.

O local do diagnóstico, presença de cirrose hepática, escore APACHE II, nível de lactato arterial, número de disfunção nos órgãos e necessidade de ventilação mecânica foram associados independente ao aumento do risco de mortalidade hospitalar entre pacientes com sepse grave e choque séptico. Aumento da idade, local do diagnóstico, APACHE II escore necessidade de ventilação mecânica e administração de vasopressores foram associados independentemente ao risco aumentado de mortalidade hospital entre os pacientes idosos.

## DISCUSSÃO

Este estudo demonstrou que as taxas de mortalidade hospitalar e do 28º dia não apresentaram diferença entre pacientes idosos e não idosos submetidos à terapia precoce guiada por metas para sepse aguda e choque séptico. No entanto, o envelhecimento foi um preditor independente da mortalidade hospitalar entre idosos. As metas terapêuticas nas primeiras 6 horas de ressuscitação, a partir do diagnóstico inicial de sepse aguda e choque séptico, foram alcançadas de forma similar nos dois grupos. Porém, administrou-se menos fluído para estabilização hemodinâmica nos pacientes idosos.

Geralmente pacientes idosos recebem tratamento menos intensivo comparado com não idosos, provavelmente devido possibilidade de efeitos deletérios de uma terapia agressiva e preocupação de sobrecarga de fluído.^(^ ^16^ ^)^ Recentemente, o aumento da aceitação de intervenções complexas na UTI em pacientes idosos esteve associado ao tratamento mais intensivo e à melhora na sobrevida.^(^ ^10^ ^)^ Talvez tal resultado se dê devido a maior experiência na assistência a pacientes idosos ao longo dos anos e pelos avanços técnicos, como protocolos associados com ferramentas de monitoria hemodinâmica de terapia de substituição renal, representando, portanto, a verdadeira evolução na prática ao longo dos tempos. Os resultados do nosso estudo confirmam esses achados. A proporção de pacientes idosos recebendo ventilação mecânica e vasopressora não mostrou diferença comparada a de pacientes mais jovens.

Nossos achados confirmam a tolerância de pacientes idosos para algoritmo de terapia guiada por metas para ressuscitação de sepse aguda e choque séptico, não mostrando diferença na mortalidade e comparado a pacientes não idosos. Esses resultados têm importantes implicações clínicas devido à existência de um aumento na demanda de internações na UTI de pacientes idosos, o que é frequentemente associado com alto custo e disponibilidade global limitada de leitos na UTI.^(^ ^7^ ^,^ ^17^ ^,^ ^18^ ^)^ Além disso, os resultados do nosso estudo apoiam o conceito de que a internação em UTI e a implementação da terapia precoce guiada por metas não deve ser negada a pacientes idosos com sepse aguda e choque séptico.

O impacto da idade em altas taxas de mortalidade devido à sepse não é observado uniformemente em estudos epidemiológicos.^(^ ^11^ ^,^ ^19^ ^,^ ^20^ ^)^ Outras análises retrospectivas demostraram que a idade foi associada ao aumento significativo do risco de óbito em pacientes idosos com sepse aguda e choque séptico.^(^ ^11^ ^,^ ^18^ ^)^ Todavia, esses estudos são baseados em base de dados para diagnóstico de sepse, o que pode apresentar erros e falta de aspectos importantes, tais como aderência ao tratamento proposto. Em nosso estudo, de forma similar, após ajustes nas características de linha de base dos pacientes em modelo de regressão logística multivariada, a idade foi associada de maneira independentemente associada com aumento do risco de mortalidade hospitalar em pacientes idosos com sepse aguda e choque séptico.

A maioria dos estudos abordando a mortalidade em pacientes com sepse tem focado em resultados de curto prazo.^(^ ^4^ ^,^ ^12^ ^)^ Poucos estudos observacionais tem analisado o prognóstico de longo prazo de pacientes idosos com sepse aguda e choque séptico naqueles submetidos a terapia precoce guiada por metas. Lemay et al. relataram taxa de mortalidade de 1 ano de 31% em idosos com sepse.^(^ ^20^ ^)^ Porém, a aderência a metas terapêuticas específicas, além de administração de antibióticos, não foi relatada, e o estudo contou com base de dados administrativos para diagnóstico de sepse. Wang et al. também descreveram taxa de mortalidade em 1 ano de 23% em população de pacientes adultos com idade de 45 anos ou mais, com sepse definida como hospitalização ou tratamento na unidade de emergência para infecções agudas com presença de dois ou mais critérios de resposta inflamatória sistêmica, sem mencionar pacientes da UTI com sepse aguda e choque séptico.^(^ ^21^ ^) ^ Recentemente, demostrou-se que a sobrevida de longo prazo em pacientes com falha circulatória (incluindo sepse) é deficiente, com taxa de mortalidade de 92 e 97% após 6 e 12 meses, respectivamente.^(^ ^13^ ^)^ Esses achados apoiam a hipótese de que a mortalidade a longo prazo persiste entre aqueles pacientes com sepse, provavelmente devido aos ativadores da sepse e um processo patofisiológico independente, levando a morte precoce.

Infecções respiratórias representaram a maioria dos casos de sepse em pacientes idosos, enquanto infecções abdominais foram a causa mais comum em pacientes jovens. Um achado que não tem sido confirmado por outros autores é a infecção respiratória como a principal fonte de infecções em pacientes idosos e não idosos.^(^ ^11^ ^) ^Uma possível explicação para os achados interessantes terem maior incidência de cirrose hepática e transplante de órgãos sólidos em pacientes jovens provavelmente reflete a alta proporção de pacientes com peritonite bacteriana espontânea.

Nosso estudo apresenta limitações. Primeiro, não foi possível avaliar o estado funcional antes e depois da alta da UTI. O estado funcional tem relatado fatores preexistentes e tem grande representatividade comparada com a idade nos resultados apresentados, incluindo pacientes idosos e não idosos.^(^ ^22^ ^) ^Segundo, o estudo foi realizado em único centro com abordagem retrospectiva, que limita potencialmente a generalização dos achados. Por fim, foi utilizada a idade de corte de 65 anos, conforme definição da Organização Mundial da Saúde. Porém, conforme mencionado, a idade nem sempre é representativa da condição funcional dos pacientes.

## CONCLUSÃO

Na população de pacientes idosos e não idosos com sepse aguda e choque séptico, a ressuscitação precoce dos pacientes idosos não se associou ao aumento da mortalidade. Pacientes idosos com sepse aguda ou choque séptico podem se beneficiar de ressuscitação agressiva e tratamento com modalidade mais avançadas. No entanto, sugerem-se novos estudos prospectivos, que abordem o impacto ao longo prazo das manobras de ressuscitação no estado funcional e qualidade de vida nessa população.

**Tabela 4:** Análise de regressão logística univariada e multivariada abordando principais fatores de risco para mortalidade hospitalar, incluindo 848 pacientes com sepse aguda e choque séptico

Características	Análise univariada			Análise multivariada		
	OR	IC95%	Valor de p	OR	IC95%	Valor de p
Sexo masculino	0,82	0,61-1,10	0,182	0,68	0,48-0,97	0,032
Local do diagnóstico						
Unidade de emergência	1,00					
Ambulatório	3,10	2,14-4,48	<0,001	2,74	1,78-4,22	<0,001
Assistência intermediária	4,93	3,15-7,71	<0,001	4,39	2,63-7,35	<0,001
Unidade de terapia intensiva	5,78	3,59-9,31	<0,001	3,64	2,04-6,50	<0,001
Cirrose hepática	2,62	1,65-4,18	<0,001	2,13	1,22-3,72	0,008
Infecção do trato urinário	0,30	0,18-0,49	<0,001	0,40	0,21-0,75	0,040
Escore APACHE II	1,11	1,08-1,13	<0,001	1,08	1,05-1,11	<0,001
Lactato arterial	1,18	1,12-1,25	<0,001	1,14	1,06-1,22	<0,001
Número de disfunções de órgãos	1,56	1,38-1,76	<0,001	1,21	1,04-1,40	0,012
Ventilação mecânica	4,94	3,62-6,73	<0,001	1,89	1,29-2,78	0,001

APACHE II: *Acute Physiology and Chronic Health Evaluation II* (escore pode variar de zero a 71, com alto escore indicando doença mais aguda). OR: *odds ratio*; IC95%: intervalo de confiança de 95%.

**Tabela 5:** Análise de regressão logística univariada e multivariada abordando principais fatores de risco para mortalidade hospitalar incluindo 531 pacientes idosos (≥65 anos)

Características	Análise univariada			Análise multivariada		
	OR	IC95%	Valor de p	OR	IC95%	Valor de p
Idade, anos	1,04	1,02-1,07	<0,001	1,04	1,01-1,07	0,003
Sexo masculino	0,69	0,48-1,00	0,048	0,61	0,40-0,94	0,023
Local do diagnóstico						
Unidade de emergência	1,00					
Enfermaria	2,71	1,67-4,39	<0,001	2,40	1,37-4,20	0,002
Assistência intermediária	3,82	2,31-6,30	<0,001	3,71	2,07-6,65	<0,001
Unidade de terapia Intensiva	4,12	2,31-7,37	<0,001	3,07	1,52-6,20	0,002
Hipertensão sistêmica	0,63	0,44-0,90	0,012	0,56	0,36-0,86	0,009
*Diabetes mellitus *	0,58	0,39-0,87	0,008	0,58	0,36-0,94	0,025
Infecção abdominal	0,69	0,40-1,18	0,174	0,50	0,27-0,95	0,034
Escore APACHE II	1,09	1,06-1,12	<0,001	1,06	1,03-1,10	<0,001
Ventilação mecânica	5,11	3,47-7,52	<0,001	2,18	1,36-3,52	0,001
Administração de vasopressor	3,25	2,17-4,85	<0,001	1,98	1,24-3,18	0,005

APACHE II: *Acute Physiology and Chronic Health Evaluation II *(escore pode variar de zero a 71, com alto escore indicando doença mais aguda). OR: *odds ratio*; IC95%: intervalo de confiança de 95%.
